# Analysis of gene expression patterns modulated by tuberculous pleural effusion–derived exosomal miRNAs in lung cancer

**DOI:** 10.3389/fgene.2026.1828734

**Published:** 2026-05-29

**Authors:** Goeun Park, Hyun-Jung Kang, Yoonki Hong, Jae Jun Lee, Seung-Ho Shin, Ji Young Hong

**Affiliations:** 1 Institute of New Frontier Research Team, Hallym University College of Medicine, Chuncheon, Republic of Korea; 2 Department of Internal Medicine, School of Medicine, Kangwon National University, Kangwon National University Hospital, Chuncheon, Republic of Korea; 3 Chuncheon Artificial Intelligence Center, Chuncheon Sacred Heart Hospital, Chuncheon, Republic of Korea; 4 Department of Biomedical Informatics, Chuncheon Sacred Heart Hospital, Chuncheon, Republic of Korea; 5 Department of Internal Medicine, Division of Pulmonary, Allergy and Critical Care Medicine, Chuncheon Sacred Heart Hospital, Hallym University Medical Center, Chuncheon, Gangwon-do, Republic of Korea

**Keywords:** lung cancer, miRNA regulation, miRNA–mRNA network, functional analysis, tuberculosis

## Abstract

**Introduction:**

Previous pulmonary tuberculosis (TB) is a known risk factor for lung cancer. Earlier studies have demonstrated that tuberculous pleural effusion (TPE)-derived exosomal miRNAs are involved in lung cancer progression. This study aimed to identify potential miRNA–mRNA regulatory pathways contributing to the pathogenesis of TB-associated lung cancer.

**Methods:**

We isolated Exosomes from the lung effusions of patients with TB and injected intratumorally into lung cancer xenograft mice to model the TB–lung cancer interaction. To identify TB-associated regulators relevant to lung cancer, we first examined differentially expressed miRNAs (DEMs) in exosomes from patients with TB. In parallel, we identified differentially expressed genes (DEGs) in xenograft lung cancer following injection with TB-derived exosomes and analyzed their interactions with the DEMs. Network analysis was then applied to interpret miRNA-mRNA regulatory relationships, with TB-related sub-networks selected for further study.

**Results:**

In total, five DEMs and 54 DEGs were identified. Pathway enrichment analysis indicated that these DEGs were linked to oxidative phosphorylation, ribosome biogenesis, mitochondrial adenosine triphosphate synthesis, and NADH dehydrogenase activity. Further network analysis with Cytoscape revealed a potential miRNA–mRNA regulatory network encompassing 94 genes expanded from the selected 54 DEGs and 4 DEMs. Finally, *in vitro* assays validated that the identified cancer-related genes (*NDUFA4*, *RPS27*, and *COX7A2*) are involved in regulating miRNA expression.

**Conclusion:**

In conclusion, our findings suggest potential indirect links between TB-derived exosomal miRNAs and lung cancer associated genes. These results provide a preliminary regulatory framework, and while further functional validation is warranted, they offer exploratory insights into the molecular landscape of TB-associated lung cancer.

## 1 Introduction

Tuberculosis (TB) affects nearly a quarter of the world’s population, with 1.3 million deaths reported in 2020 alone (World Health Organization, 2021). Lung cancer likewise remains a leading cause of global mortality, with the International Agency for Research on Cancer estimating about 1.8 million deaths each year (Bray et al., 2024). Both diseases impose significant socioeconomic burdens due to their high rates of mortality and morbidity. Several epidemiological studies have also highlighted a correlation between TB and lung cancer (Abdeahad et al., 2022; Cabrera-Sanchez et al., 2022; Liao et al., 2023).

Dacosta and Kinare (1991) reported that approximately 30% of patients with bronchogenic carcinoma present with either active TB or residual tuberculous scars in the lung parenchyma, compared with only 7% in the general population. Independent of cigarette smoking or the presence of chronic obstructive pulmonary disease, a history of TB has been identified as an independent risk factor for lung cancer (Moon et al., 2023). Moreover, a TB history is considered a poor prognostic indicator that is associated with increased mortality in patients with lung cancer (Heuvers et al., 2012; Hong et al., 2016; Liao et al., 2023). Several studies have explored the mechanisms underlying this relationship. Nalbandian et al. (2009) demonstrated in animal models that chronic TB lung lesions create an environment conducive to carcinogenesis. More recent mouse studies showed that tuberculous fibrosis can heighten the tumorigenic potential of lung cells, potentially through NOX4-associated autophagy (Woo et al., 2021). Woo et al. further found that elevated Arg1+ M2 polarization following TB infection promotes lung cancer metastasis, suggesting that macrophages may serve as therapeutic targets in post-tuberculous lung cancer (Woo et al., 2024). Notably, both the tumor microenvironment and TB granulomas share a central feature of exhausted T-cell phenotypes, which appear to influence one another (Ashenafi and Brighenti, 2022). However, detailed studies on the molecular mechanisms and therapeutic target genes linking TB-associated lung lesions to lung cancer remain limited.

Exosomes are phospholipid bilayer vesicles secreted by various cells into the extracellular space under both homeostatic and pathological conditions (Mathivanan et al., 2010). They can be detected in bodily fluids such as pleural effusion (PE), urine, blood, and bronchoalveolar lavage (Boukouris and Mathivanan, 2015). Recent evidence indicates that exosomes transport proteins, miRNAs, and mRNAs in active form to adjacent cells or distant organs, facilitating cell-to-cell communication (Nail et al., 2023; Wang et al., 2020). Exosomes may play a role in mediating the TB–lung cancer interaction and clarifying these mechanisms could provide valuable insights into TB-associated lung cancer.

In this study, we employed next-generation sequencing and bioinformatics analysis to investigate the association between TB and lung cancer. Exosomes were isolated from the lung effusions of patients with TB and injected intratumorally into lung cancer xenograft mice to model the TB–lung cancer interaction. To identify TB-associated regulators relevant to lung cancer, we first examined differentially expressed miRNAs (DEMs) in exosomes from patients with TB. In parallel, we identified differentially expressed genes (DEGs) in xenograft lung cancer following injection with TB-derived exosomes and analyzed their interactions with the DEMs. Network analysis was then applied to interpret miRNA–mRNA regulatory relationships, with TB-related sub-networks selected for further study. Our work centers on miRNAs in TB-derived exosomes and their regulation of mRNA expression in lung cancer cells, potentially offering new insights into the mechanisms of mRNA regulation in TB-associated lung cancer.

## 2 Materials and methods

### 2.1 Patients and sample collection

PE samples were collected at Chuncheon Sacred Heart Hospital during routine thoracentesis for diagnostic purposes. All human experiments were conducted in accordance with the Declaration of Helsinki. The collection and use of these samples were approved by the hospital’s Research Ethics Committee (institutional review board number: 2012-27). All patients provided informed consent for sample collection and anonymous use of clinical information. Ten milliliters of pleural fluid were collected in sterile tubes from each patient via thoracentesis. Samples were centrifuged at 3,000 × g for 10 min, and the supernatants were stored at −80 °C until analysis. PEs were classified as exudative or transudative according to Light’s criteria (Light et al., 1972). Tuberculous effusion was defined as pleural fluid that was culture-positive for *Mycobacterium tuberculosis* and met the criteria for exudative PE. Transudative PE served as the control group.

### 2.2 Exosome isolation and quantification

Briefly, exosomes were isolated using an Exo2D-EV isolation kit (Exosome Plus, Inc., Seoul, Republic of Korea) according to the manufacturer’s protocol. After centrifugation of 5 mL samples at 3,000 × g for 15 min to remove debris, the supernatant was incubated with Exo2D solution at 4 °C for 30 min, followed by centrifugation at 3,000 × g for 30 min. The resulting exosome pellet was resuspended in phosphate-buffered saline for downstream analyses. Vesicles were operationally defined as exosomes based on nanoparticle tracking analysis, transmission electron microscopy, and expression of canonical exosomal markers. Nanoparticle tracking analysis (NTA) was performed to determine particle size distribution and concentration (particles/mL) using the NanoSight NS300 system (Malvern Panalytical, Malvern, Worcestershire, UK). EV samples were resuspended in sterile, filtered PBS to generate a dilution in which 20–120 particles/frame were tracked; for each sample, five recordings of 60 s were performed (for a total of 1,498 frames) and were captured and analyzed using NTA 3.1 software by applying optimized settings. Data are presented as the mean of five recordings. A549 cells treated with TPE-derived exosomes or transudate-derived exosomes for 48 h were examined by field emission transmission electron microscopy (FE-TEM, JEM 2100F; JEOL, Tokyo, Japan). Exosome samples were placed on carbon-coated copper grids and negatively stained with 2% uranyl acetate. Images were acquired at an accelerating voltage of 200 kV. Exosomal protein concentration was determined using a bicinchoninic acid protein assay (Thermo Fisher Scientific) prior to experimental use. For Western blot analysis, 30 μg of protein from A549 cells and exosomal lysates were lysed using RIPA buffer supplemented with a protease inhibitor cocktail (GenDEPOT, Baker, TX, United States). Proteins were transferred onto membranes, which were subsequently incubated overnight at 4 °C with the following primary antibodies: β-actin, CD63, and CD81 (Abbkine, Atlanta, GA, United States), and CD9 (LifeSpan BioSciences, Shirley, MA, United States). After washing with PBS containing Tween-20, the membranes were incubated with appropriate horseradish peroxidase–conjugated secondary antibodies. Protein signals were visualized using enhanced chemiluminescence (ECL) detection reagents (Thermo Fisher Scientific, Waltham, MA, United States). Total RNA was extracted from the purified exosomes using TRIzol reagent (Thermo Fisher Scientific, Waltham, MA, United States) according to the standard protocol. This RNA was used for subsequent RNA analysis and sequencing.

### 2.3 Xenograft model

For xenograft generation, 4 × 10^6^ A549 cells were subcutaneously injected into the flanks of 10 male athymic nude mice (six to eight weeks old; Charles River, Lecco, Italy). Mice were randomly assigned to three groups: a vehicle control group receiving PBS (n = 3), a low-dose group receiving 100 µg of TPE-derived exosomes (n = 5), and a high-dose group receiving 200 µg of TPE-derived exosomes (n = 2). Exosome input was quantified based on total protein concentration (µg), and consistency with particle number was verified by nanoparticle tracking analysis. TPE-derived exosomes were administered via intratumoral injection in 100 µL PBS every 2 days for a total of seven injections. Each mouse was considered an experimental unit, and tumor growth was measured individually. Investigators responsible for tumor measurement and data analysis were blinded to group allocation throughout the study. Mice were maintained according to the United Kingdom Co-ordinating Committee on Cancer Research guidelines and housed in a specific pathogen-free (SPF) facility under controlled conditions (temperature 22 °C ± 2 °C, relative humidity 50% ± 10%, and a 12-h light/dark cycle). They were kept in individually ventilated cages (IVCs, 4–5 animals per cage) with free access to standard rodent chow and autoclaved tap water *ad libitum*. Environmental enrichment, including nesting material and shelters, was provided. Tumor volumes were measured every other day with calipers and calculated as (tumor length × width^2^)/2. Seven days after transplantation, when the tumors reached approximately 40 mm^3^, TPE–derived exosomes were injected intratumorally in 100 μL PBS at doses of 100 μg or 200 μg per mouse every 2 days, for a total of seven injections. Humane endpoints included >10% with morbidity, Body Condition Score ≤2/5, impaired mobility, dehydration, respiratory distress, or moribund state. For tumor studies, mice were euthanized if tumor size reached 1.5 cm in any dimension, ulcerated/infected, or interfered with normal behavior. On day 21, mice were euthanized under isoflurane inhalation anesthesia (3%–5% for induction and 1.5%–2.5% for maintenance in oxygen) followed by cervical dislocation, and tumor tissues were dissected for analysis. RNA from xenograft tissues was subjected to quantitative real-time polymerase chain reaction (qRT-PCR) to evaluate the effects of TPE–derived exosomes on A549 cells. The study protocol was prepared and approved by the Institutional Animal Care and Use Committee (IACUC) of Hallym University (No: Hallym 2020-21) and was duly registered with the committee, as this was a preclinical study. All experiments were conducted in accordance with the ARRIVE guidelines.

### 2.4 Generation of sequencing data

Total RNA was extracted and purified for subsequent miRNA library preparation. Exosomal RNA, including miRNA, was isolated using the miRNeasy Mini Kit (Qiagen, Hilden, Germany). miRNA libraries were prepared with the SMARTer smRNA-seq Kit (Takara Bio United States, San Jose, CA, United States), specifically designed for efficient and accurate generation of small RNA sequencing libraries from purified RNA samples. This kit employs Switching Mechanism at the 5′end of RNA Template (SMART) technology to ensure high-quality, comprehensive library construction. The miRNA libraries were sequenced on an Illumina platform using single-end reads with a length of 51 bp. For mRNA expression profiling, sequencing libraries were prepared with the MGIEasy RNA Directional Library Prep Kit (MGI Tech Co., Ltd., Shenzhen, China), which enables strand-specific library generation and preserves transcript directionality. The preparation process included RNA fragmentation, reverse transcription, adapter ligation, and PCR amplification. Following library construction, the mRNA libraries were sequenced on the MGISEQ system, generating 150 bp paired-end reads. The sequencing output and quality metrics for all libraries are summarized in Supplementary Table 1.

### 2.5 Raw data processing and identification of DEGs and DEMs

Two different bioinformatic pipelines were applied to obtain miRNA and mRNA expression profiles. For miRNA expression profiling, FastQC (v0.11.7) and Cutadapt (v2.8) (Martin, 2011) were used to filter out low-quality reads and adapter sequences from the raw sequencing data. miRNA expression profiling was performed using Bowtie (v1.1.2) (Langmead et al., 2009) and miRDeep2 software (v2.0.0.8) (Friedländer et al., 2012). Sequencing reads were aligned to reference miRNA sequences in miRBase (v22.1) (Kozomara et al., 2019) and the resulting read counts were analyzed using the EdgeR pipeline (v4.4.0) (Robinson et al., 2010). Raw count data generated from miRDeep2 were filtered to remove low-abundance miRNAs using the filterByExpr function. Library size normalization was performed using the trimmed mean of M-values (TMM) method. Count data were modeled using a negative binomial distribution, and dispersion parameters were estimated using empirical Bayes methods. A generalized linear model (GLM) was fitted to the data, and differential expression was assessed using likelihood ratio tests. miRNAs with a fold change ≥2 and a false discovery rate (FDR) < 0.05, as determined using the Benjamini–Hochberg correction, were considered differentially expressed. For mRNA expression profiling, strand-specific (first-strand) RNA-seq reads were preprocessed to remove low-quality bases and adapter sequences using Skewer (v0.2.2) (Jiang et al., 2014). Cleaned reads were aligned to the human reference genome (hg38) using STAR (v2.5) with “--outFilterIntronMotifs RemoveNoncanonicalUnannotated” parameter (Dobin et al., 2013). Gene expression levels were estimated using Cuffquant in Cufflinks package (v2.2.1) (Trapnell et al., 2010) and normalized as fragments per kilobase of transcript per million mapped reads (FPKM). Differential expression analysis was performed using Cuffdiff from the Cufflinks package with the “--library-type fr-firststrand” parameter (Trapnell et al., 2013). Cuffdiff estimates expression variability using a beta-negative binomial model and performs internal normalization to account for library size and transcript length. Genes with a fold change ≥2 and a false discovery rate (FDR) < 0.05 (Benjamini–Hochberg correction) were considered differentially expressed.

### 2.6 GO and KEGG pathway enrichment analysis of DEGs

GO and KEGG pathway enrichment analyses were performed to identify functional genes using ClusterProfiler (v4.14.6) in R software (Yu, 2024). Differentially expressed genes were converted to Ensembl and Entrez identifiers using bitr, and over-representation analysis was conducted using enrichGO and enrichKEGG. Enrichment significance was evaluated using a hypergeometric test, and P-values were adjusted for multiple testing using the Benjamini–Hochberg method. Significant GO terms were categorized into three functional groups: biological process, molecular function, and cellular component. Only GO and KEGG terms with a significance level of FDR <0.01 were included in subsequent analyses.

### 2.7 Interaction network analysis

Network analysis was performed using Cytoscape software (Shannon et al., 2003) to examine the interactions between DEGs and DEMs. To explore the network between the selected DEGs and DEMs, we first expanded the interaction network. Using CluePedia (Bindea et al., 2013), we utilized Activation, Inhibition, and Expression data from the STRING database v11.0 (Szklarczyk et al., 2019) to select 40 additional genes highly associated with the DEGs. The significance of these genes was then evaluated using the ‘Hub enrichment’ rule within CluePedia. The top 40 genes, ranked based on their involvement in enriched interactions within the network, were included to expand the analysis. Predicted interactions between DEGs and DEMs were retrieved from the miRDB database v6.0, which is based on miRBase v22 annotations (Chen and Wang, 2020). To ensure high-confidence predictions, only DEGs-DEMs interactions with a miRDB prediction score ≥80 were retained.

### 2.8 Cell migration and invasion assays

Cell migration and invasion assays were performed as previously described (Kang et al., 2024). For the wound healing assay, A549 cells (5 × 10^5^) were seeded in 6-well plates and cultured to near confluence. A linear scratch was created using a sterile pipette tip, and cells were incubated with TPE-derived or transudate-derived exosomes. Images were captured at 0, 24, and 48 h using an inverted microscope, and migration was quantified as the percentage of wound closure using ImageJ software. Transwell invasion assays were performed using inserts with 8 μm pores. The upper surface of the inserts was coated with Matrigel diluted 1:50 in serum-free medium and incubated at 37 °C for 1 h to allow gel formation. A549 cells were seeded in the upper chamber, while exosomes were added to the lower chamber. After incubation for 24 h, non-invading cells on the upper surface were removed, and invaded cells on the lower surface were fixed and stained with 0.1% crystal violet. Stained cells were counted in randomly selected fields under a light microscope. Data are presented as the average number of invaded cells per field from at least three independent biological replicates.

### 2.9 *In vitro* miRNA transfection experiment

The human adenocarcinoma cell line A549 was purchased from the American Type Culture Collection (ATCC, Manassas, VA, United States) and cultured according to the manufacturer’s instructions. A549 cells were maintained in RPMI 1640 medium (BYLABS, Hanam, Republic of Korea) supplemented with 10% fetal bovine serum, 100 U/mL penicillin, and 100 μg/mL streptomycin at 37 °C in a humidified incubator with 5% CO2. To investigate the correlation between miRNAs from TPE-derived exosomes and gene expression in lung cancer cells, A549 cells were transfected with miRNA mimics or inhibitors for hsa-miR-1290, hsa-miR-185, hsa-miR-30a, and hsa-miR-224 (200 pM) (Bioneer, Daejeon, Republic of Korea). Transfections were performed using AccuFectTM transfection reagent (Bioneer) according to the manufacturer’s protocol, and transfection efficiency was assessed by RT-PCR. miRNAs were extracted from A549 cells treated with mimics or inhibitors using the miRNeasy® Mini Kit (Qiagen) and quantified by optical density at 260 nm. cDNA was synthesized using the Mir-X™ miRNA First-Strand Synthesis Kit (Clontech Laboratories, Inc., Mountain View, CA, United States). Total RNA was extracted from A549 cells with easy-BLUE reagent (iNtRON Biotechnology, Seongnam-si, Korea), and first-strand cDNA was synthesized using the Maxime RT PreMix Kit (iNtRON Biotechnology) according to the manufacturer’s instructions. RT-PCR was carried out on the qTOWER3 G system using SYBR Green I as the double-stranded DNA–specific dye. PCR amplification consisted of 40 cycles of denaturation at 94 °C for 15 s and annealing at 60 °C for 1 min. Relative expression levels were calculated using the 2^-ΔΔCt^ method, and data were normalized to β-actin expression in each sample relative to the control group. Primer sequences for each gene are listed in Supplementary Table 2.

### 2.10 Statistical analysis

All statistical analyses were performed using GraphPad Prism software v8.02 (GraphPad Software Inc., San Diego, CA, United States) and home-built scripts in R software v4.4.2 (R Core Team, 2024). Data are presented as the mean ± standard error of the mean (SEM). Expression analyses from the xenograft experiments and RT-PCR were compared to the control group using Student’s t-test, and a P-value <0.05 was considered statistically significant. The identification of DEGs and DEMs, as well as functional analyses, were conducted using the aforementioned software. To correct for multiple testing, the false discovery rate was controlled using the Benjamini–Hochberg method. Significant DEGs and DEMs were selected based on an FDR <0.05, while significant Gene Ontology (GO) terms and KEGG pathways were selected based on an FDR <0.01.

## 3 Results

### 3.1 Identification of DEMs in patients with TB

**FIGURE 1:**
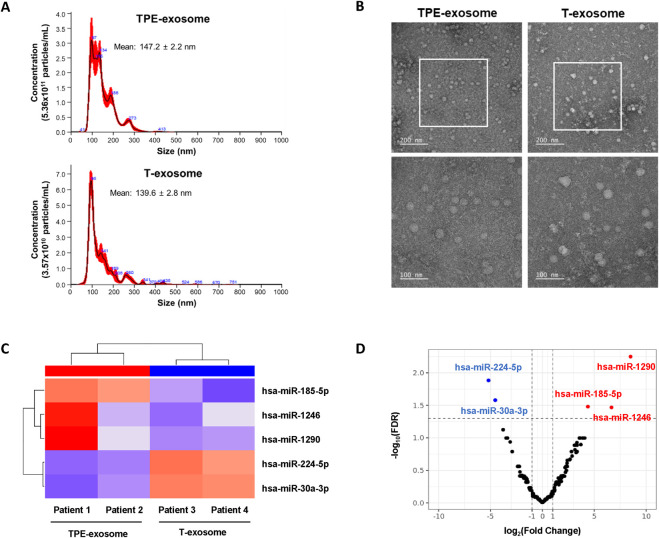
Differentially expressed miRNAs (DEMs) identified in TPE-derived exosomes. **(A)** NTA of exosomes isolated from TPE and T. The size distribution of extracellular vesicles was measured using NTA, showing a predominant peak at approximately 147.2 nm (TPE) and 139.6 nm (T). Particle concentration was estimated at 5.36 × 10^11^ (TPE) and 3.57 × 10^10^ (T) particles/mL. **(B)** Transmission electron microscopy (TEM) images of exosomes isolated from TPE and T. Scale bars: 200 nm (top panel) and 100 nm (bottom panel). **(C)** Heatmap showing miRNAs that were significantly altered (Fold change ≥2, FDR <0.05, Benjamini–Hochberg correction) in TPE-derived exosome compared to T-derived exosome. Darker red indicates higher expression, while darker blue indicates lower expression. **(D)** Volcano plot showing differentially expressed miRNAs (DEMs) between TPE-derived and T-derived exosome. Dashed lines denote the thresholds for defining DEG candidates: an absolute 2-fold change on the x-axis and FDR of 0.05 on the y-axis. Red and blue dots represent significantly upregulated and downregulated miRNAs, respectively, while black dots indicate non-significant miRNAs. Selected DEMs are labeled. DEMs, differentially expressed miRNAs; TPE, tuberculous pleural effusion; T, transudate; NTA, Nanoparticle tracking analysis.

To identify miRNAs differentially expressed in tuberculous PE (TPE)-derived exosomes, we isolated exosomes from two tuberculous effusions and two transudative PEs (Table 1). Tuberculous effusion was defined as pleural fluid culture positive for *M. tuberculosis*, while transudative PEs served as the reference control group. Nanoparticle tracking analysis (NTA) demonstrated that TPE-derived exosomes and transudate-derived exosomes had comparable size distributions, with mean diameters of 147.2 ± 2.2 nm and 139.6 ± 2.8 nm, respectively (Figure 1A). The presence of exosomes isolated from pleural fluid was further confirmed by transmission electron microscopy (Figure 1B) and by Western blot analysis showing the expression of canonical exosomal markers, including CD63, CD9, and CD81 (Supplementary Figure 1). Comparative miRNA profiling of exosomal RNA between the two groups revealed five significant DEMs (Fold change ≥2, FDR <0.05, Benjamini–Hochberg correction). The identified DEMs were hsa-miR-30a-3p, hsa-miR-224-5p, hsa-miR-1246, hsa-miR-185-5p, and hsa-miR-1290, with three upregulated and two downregulated in patients with TB (Figures 1C,D; Supplementary Table 3). These DEMs were then used in downstream analyses to explore potential regulatory associations with DEGs implicated in lung cancer proliferation.

**TABLE 1:** Characteristics of patients with pleural effusion.

Characteristics	Tuberculous effusion 1	Tuberculous effusion 2	Transudate 1	Transudate 2
Age (years)	77	23	51	51
Sex	Male	Male	Male	Male
Comorbidity	HTN, IHD	None	Cirrhosis, CRF	WE
Pleural fluid analysis				
Total protein (g/dL)	4.7	5.4	3	<2.0
Albumin (g/dL)	2.5	3.6	1.8	1.2
LDH (IU/L)	178	1,613	76	61
CRP (mg/L)	6.2	6.7	<4.0	4.8
ADA (IU/L)	77.5	102.5	29	6
Serum				
Total protein (g/dL)	6.4	7.1	6.5	5.2
Albumin (g/dL)	3.4	4.6	3.6	3.1
LDH (IU/L)	229	225	176	177
Light criteria				
Protein ratio (pleural/serum) > 0.5	Yes	Yes	No	No
LDH ratio (pleural/serum) > 0.6	Yes	Yes	No	No
Pleural LDH >2/3 of serum LDH	Yes	Yes	No	No
Positive pleural fluid TB culture	Yes	Yes	No	No

HTN, hypertension; IHD, ischemic heart disease; CRF, chronic renal failure; WE, Wernicke’s encephalopathy; LDH, lactate dehydrogenase; CRP, C-reactive protein; ADA, adenosine deaminase; TB, tuberculosis.

### 3.2 Differential gene expression and functional enrichment analysis in TB exosome-treated lung cancer xenografts

**FIGURE 2:**
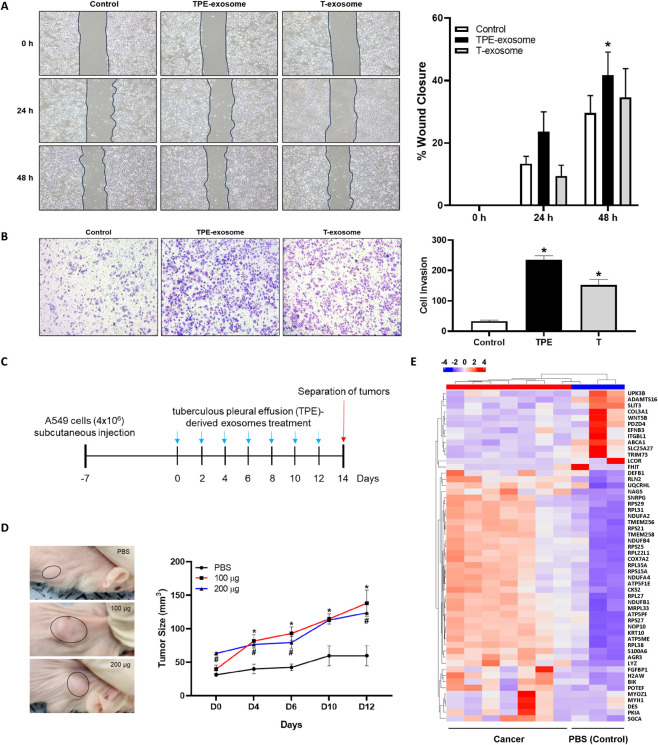
Effects of TPE-derived exosomes on lung cancer progression and associated differentially expressed genes (DEGs). **(A)** Wound healing assay showing the effect of TPE and T-derived exosomes on the migratory capacity of A549 cells. Wound closure was quantified at the indicated time points. *p < 0.05 vs. control. **(B)** Transwell invasion assay showing the effect of TPE and T-derived exosomes on A549 cell invasion. Invaded cells were stained and quantified after 24 h *p < 0.05 vs. control. **(C)** Schematic overview of the experimental design used to identify DEGs associated with TPE-derived exosome-mediated tumor development. **(D)** Tumor growth in xenograft models measured at indicated time points following cell implantation. **(E)** Heatmap analysis between TPE-derived exosome-treated mice and PBS-injected control mice. ‘#’ denotes a statistically significant difference compared with the PBS control group at 100 μg TPE-derived exosome treatment, and ‘*’ denotes a significant difference compared with the PBS control group at 200 μg TPE-derived exosome treatment. Data are presented as mean ± standard error of the mean DEGs, differentially expressed genes; TPE, tuberculous pleural effusion; PBS, phosphate-buffered saline.

To investigate TB-associated miRNAs that may influence lung cancer proliferation, we evaluated the effects of TPE-derived exosomes on lung cancer cell migration and invasion *in vitro* and performed *in vivo* xenograft experiments using lung cancer cells treated with exosomes derived from patients with tuberculous pleural effusion (TPE). In the wound healing and Transwell assays, TPE-derived exosomes significantly promoted the migration and invasion of lung cancer cells compared with untreated controls (Figures 2A,B). In contrast, transudate-derived exosomes did not significantly enhance lung cancer cell invasion. The *in vivo* experimental design is summarized in Figure 2C. Tumor volume measurements showed that xenografts in the TPE-treated group were significantly larger than those in the control group (TPE: 87.6 ± 16.04 mm^3^; Control: 28.5 ± 3.07 mm^3^; P < 0.05), regardless of the TPE dosage (100 μg and 200 µg) (Figure 2D). No trend in cancer proliferation according to TPE dosage was observed.

Transcriptome profiling of 7 TPE-treated xenografts and three untreated controls identified a total of 54 DEGs, including 41 upregulated and 13 downregulated genes (Fold change ≥2, FDR <0.05, Benjamini–Hochberg correction) (Figure 2E; Supplementary Table 4). Functional annotation of these DEGs was carried out using Gene Ontology (GO) and Kyoto Encyclopedia of Genes and Genomes (KEGG) enrichment analyses to clarify their potential biological roles (FDR <0.01, Benjamini–Hochberg correction).

**FIGURE 3:**
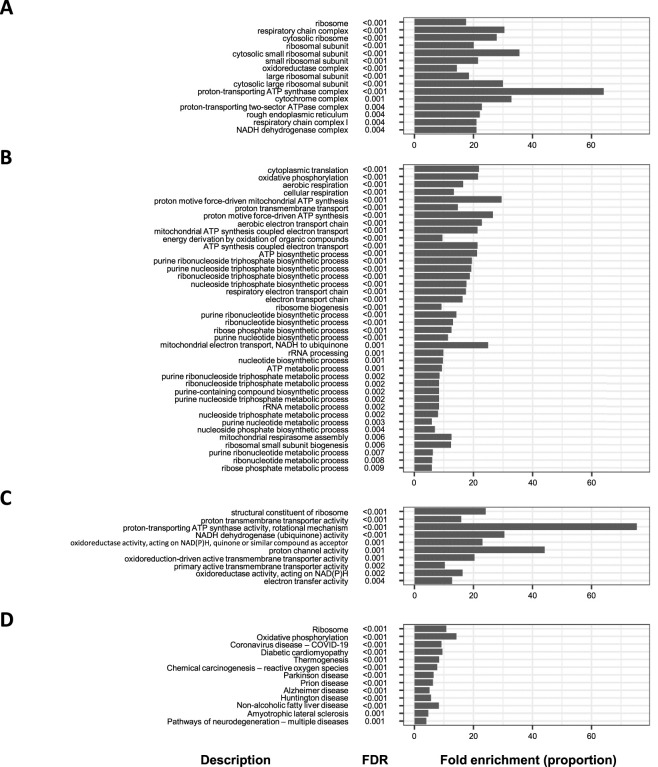
Enrichment analysis of DEGs in GO terms and KEGG pathways. Enrichment analyses were performed using the clusterProfiler package, and significantly enriched GO terms and KEGG pathways were selected based on the FDR <0.01 (Benjamini–Hochberg correction). GO enrichment results were categorized into functional groups: **(A)** cellular component, **(B)** biological process, and **(C)** molecular function. **(D)** KEGG pathways significantly enriched relative to background gene frequency. DEGs, differentially expressed genes; GO, Gene Ontology; KEGG, Kyoto Encyclopedia of Genes and Genomes.

GO enrichment analysis showed that, within the cellular component category, DEGs were significantly enriched in the ribosome, respiratory chain complex, cytosolic ribosome, ribosomal subunit, cytosolic small ribosomal subunit, and proton-transporting adenosine triphosphate (ATP) synthase complex (Figure 3A). In the biological process category, DEGs were mainly associated with cytoplasmic translation, oxidative phosphorylation, aerobic respiration, proton motive force-driven ATP synthesis, mitochondrial ATP synthesis coupled to electron transport, and energy derivation by oxidation of organic compounds (Figure 3B). For the molecular function category, enrichment was observed in structural constituents of the ribosome, proton transmembrane transporter activity, proton-transporting ATP synthase activity (rotational mechanism), NADH dehydrogenase (ubiquinone) activity, proton channel activity, and primary active transmembrane transporter activity (Figure 3C).

KEGG pathway enrichment analysis revealed that DEGs were significantly enriched in cancer-related pathways, particularly oxidative phosphorylation and chemical carcinogenesis–reactive oxygen species (Figure 3D). Taken together, these findings suggest that TPE-derived exosome treatment drives substantial transcriptional reprogramming in lung cancer xenografts, particularly affecting ribosome biogenesis, mitochondrial energy metabolism, and related molecular functions, which may contribute to enhanced tumor growth.

### 3.3 Integrated network analysis of DEGs and DEMs

To explore the associations between the identified DEGs and DEMs, we performed an integrated network analysis using protein–protein interaction databases and miRNA–mRNA regulatory interaction data (see Methods). The analysis incorporated 54 DEGs, 5 DEMs, and 40 top rank genes interacting with the DEGs.

The network analysis revealed several interaction clusters centered around specific miRNAs and oncogenic driver genes. miR-1290 was found to be a central regulator, showing extensive indirect interactions with members of the ribosomal protein gene family (RPS21, RPS25, RPS29) and eukaryotic initiation factors such as EIF5. Furthermore, miR-1290 maintains a regulatory link with the PRKAC family (PRKACA, PRKACB, PRKACG), which are closely associated with the NDUF gene subcomplex (NDUFA2, NDUFA4, NDUFB1, NDUFB4). miR-185, which was upregulated in our analysis, exhibited connectivity with key oncogenic hubs including RHOA and the PRKAC cluster. In contrast, downregulated miRNAs such as miR-30a-3p and miR-224 were integrated into distinct regulatory nodes. Specifically, miR-30a-3p was linked to a dense cluster of ribosomal proteins and biogenesis factors (WDR43, UTP20, NOL11), while miR-224 showed a direct interaction path toward COX7A2 via LCOR.

Together, these findings suggest that interactions between the identified DEMs and DEGs may contribute to lung cancer development by modulating cancer-related genes and pathways through complex regulatory networks involving both mRNAs and miRNAs.

### 3.4 *In vitro* identification of miRNA regulated cancer proliferation

**FIGURE 4:**
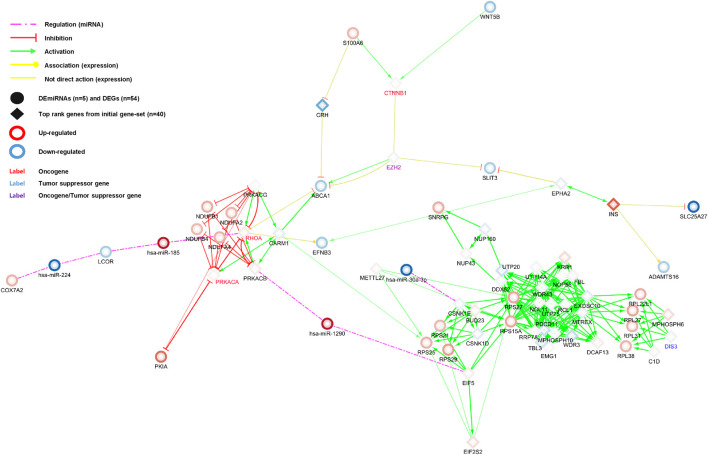
Integrated analysis of DEGs and putative miRNA regulation. DEGs identified from tumors induced by TPE-derived exosomes and DEMs were integrated using the CluePedia package. The analysis included 54 DEGs, 5 DEMs, and the top 40 ranked genes selected by enrichment analysis in CluePedia. miRNA–mRNA regulatory interactions are indicated by pink dashed edges, while other interaction types were retrieved from the STRING database. The node borders indicate the direction of regulation by TPE-derived exosomes, where red represents upregulated genes and blue represents downregulated genes. Unconnected nodes were removed to improve the clarity of the visualization. DEGs, differentially expressed genes; DEMs, differentially expressed miRNAs; TPE, tuberculous pleural effusion.

Based on a series of bioinformatics analyses, we established a potential miRNA–mRNA regulatory network, including the miR-1290–NDUFA pathway, miR-1290–RPS pathway, miR-30a–RPS pathway, miR-185–RPS pathway, and miR-224–COX7A2 pathway (Figure 4).

**FIGURE 5:**
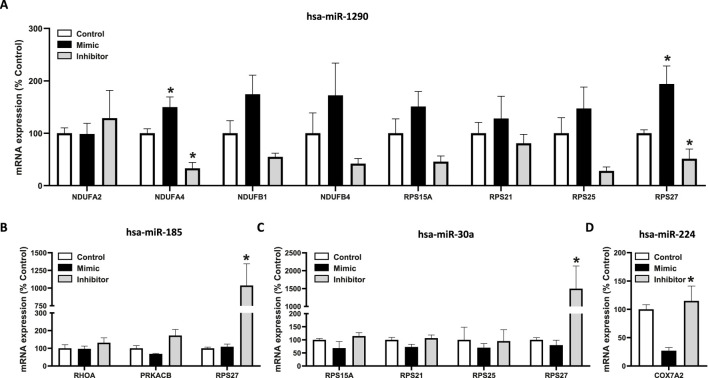
qRT-PCR validation of target gene expression following transfection with candidate miRNA mimics or inhibitors. A549 cells were transfected with sequence-specific miRNA mimics or inhibitors, with a non-targeting RNA serving as the control. Target gene expression was quantified by qRT-PCR at the indicated time points. **(A)** Expression of *NDUFA2*, *NDUFA4*, *NDUFB1*, *NDUFB4*, *RPS15A*, *RPS21*, *RPS25*, and *RPS27* 24 h after transfection with an hsa-miR-1290 mimic or inhibitor. **(B)** Expression of *COX7A2* 3 h after transfection with an hsa-miR-224 mimic or inhibitor. **(C)** Expression of *RHOA*, *PRKACB*, and *RPS27* 24 h after transfection with an hsa-miR-185 mimic or inhibitor. **(D)** Expression of *RPS15A*, *RPS21*, *RPS25*, and *RPS27* 12 h after transfection with an hsa-miR-30a mimic or inhibitor. All experiments were performed in triplicate. Data are presented as mean ± standard error of the mean (n = 3). *P* < 0.05 vs. control unless otherwise indicated. qRT-PCR, quantitative real-time polymerase chain reaction.

To validate these pathways, we transfected A549 cells with four miRNA mimics and inhibitors, respectively (Figure 5). We confirmed that the expression levels of these miRNAs were significantly altered after mimic and inhibitor transfection. In the experiment examining the indirect association between miR-1290 and ribosome biogenesis-related genes, overexpression of miR-1290 markedly increased the expression of NDUFA4, NDUFB1, NDUFB4, RPS15A, RPS21, RPS25, and RPS27. Conversely, downregulation of these genes was observed in A549 cells transfected with miR-1290 inhibitors. Significant upregulation of RPS27 was observed following the inhibition of either miR-185 or miR-30a. Compared with the negative control group, downregulation of COX7A2 was observed in the miR-224 overexpression group. These findings suggest that the miR-1290/miR-30a/miR-185–RPS27, miR-1290–NDUFA4, and miR-224–COX7A2 pathways may contribute to the pathogenesis of TB-associated lung cancer.

## 4 Discussion

During the interaction between *M. tuberculosis* and host cells, altered miRNA expression functions as a key regulatory mechanism, shaping either the pathogen’s survival strategies or the host’s defensive responses (Wang et al., 2022). Exosomal miRNAs derived from *M. tuberculosis* have been identified as important regulators of immune processes, including autophagy, apoptosis, and inflammation, thereby influencing host susceptibility and bacterial persistence (Mukhtar et al., 2024). Certain miRNAs also play central roles in inflammation, T-cell differentiation, and B-cell activation, processes closely linked to host defense as well as the pathogen’s ability to promote invasion in TB pathogenesis (Peng et al., 2022; Sinigaglia et al., 2020; Zhao Z. et al., 2019).

In our study, three upregulated DEMs (miR-1290, miR-185-5p, and miR-1246) and two downregulated DEMs (miR-30a and miR-224-5p) were identified as differentially expressed between the TPE and non-TPE groups and selected for further analysis. These findings are consistent with previous reports in the literature.

miR-185-5p has been reported as a diagnostic biomarker for TB (Wang et al., 2022). Kaushik et al. (Kaushik et al., 2021) showed that plasma exosomal miR-185 was significantly upregulated in patients with TB compared with healthy controls. Similarly, miR-1290 was identified as one of the DEMs expressed exclusively in MTB-1458–infected THP cells, compared with BCG-infected groups (Zhu et al., 2021). The miR-30 family has also been reported to undergo altered expression in patients with active TB (Wang et al., 2022). Several members, including miR-30b, miR-30c, and miR-30d, were found to be downregulated in active TB compared with healthy controls (Spinelli et al., 2017). Chen et al. (Chen et al., 2015) further demonstrated that miR-30a levels could discriminate between active TB and healthy conditions. More recent evidence showed that miR-30a regulates M. tuberculosis-induced immune responses by modulating TLR/MyD88 signaling and suppressing autophagy-mediated bacterial clearance (Wu et al., 2017). miR-224-5p has been shown to exhibit lower expression in TB-infected patients. Huang et al. (Huang et al., 2020) reported that TB-induced upregulation of circRNA-0003528 suppresses miR-224-5p, thereby activating its target CTLA4 and promoting macrophage polarization.

Previous epidemiological studies have reported an association between TB and lung cancer (Abdeahad et al., 2022; Cabrera-Sanchez et al., 2022). TB has also been directly linked to lung cancer incidence, mortality, and prognosis (Heuvers et al., 2012; Hong et al., 2016; Liao et al., 2023). Although some miRNAs are shared between TB and lung cancer, their expression patterns, target genes, and underlying molecular mechanisms in each disease remain incompletely understood (Sheikhpour et al., 2021). Kang et al. (Kang et al., 2024) demonstrated that TPE-derived exosomes promote the proliferation of lung cancer cells. However, research specifically addressing the relationship between TB-related exosomal miRNAs and cancer-related gene pathways in TB-associated lung cancer is still limited. The present study aimed to construct a potential miRNA–mRNA regulatory network involved in the interaction between TB and lung cancer.

GO and KEGG pathway enrichment analyses of DEGs from our xenograft model revealed significant involvement of ribosome biogenesis, cellular respiration, oxidative phosphorylation, and NADH dehydrogenase activity in TB-associated lung cancer. Integrated network analysis further identified several candidate miRNA–mRNA interactions (including the miR-1290–NDUFA4 axis, miR-1290–RPS27 axis, miR-30a–RPS27 axis, miR-185–RPS27 axis, and miR-224–COX7A2 axis) that may contribute to the tumorigenesis of TB-related lung cancer.

miR-1290 is aberrantly upregulated in various malignancies and functions as an oncogenic miRNA by targeting multiple tumor suppressor pathways (Sheikhpour et al., 2021). Exosomal miR-1290 has been shown to promote angiogenesis in hepatocellular carcinoma by targeting SMEK1 (Wang et al., 2021). Although the direct mechanism by which miR-1290 targets specific genes was not fully elucidated, our results indicate that miR-1290 overexpression indirectly leads to increased expression of NDUFA4 and RPS27, which are emerging as potential targets in lung cancer. This suggests a possible role for miR-1290 in promoting tumorigenesis in TB-related lung cancer.

NADH dehydrogenase one alpha sub-complex 4 (NDUFA4) is an essential component of the mitochondrial respiratory chain, associated with redox processes and ATP production (Balsa et al., 2012). It regulates the growth and metastasis of multiple cancer types by altering mitochondrial energy metabolism (Zhou et al., 2024). Notably, miR-7–mediated targeting of NDUFA4 suppresses the proliferation and metastatic potential of non-small cell lung cancer cells through modulation of key oncogenic signaling pathways such as Akt and Erk (Lei et al., 2017).

Ribosomal proteins (RPs) not only participate in ribosome biogenesis essential for cell survival but also perform extraribosomal roles in DNA repair, replication, proliferation, apoptosis, and chemoresistance (Wang et al., 2015; Zhou et al., 2015). RPS15A has been identified as a novel oncogene that prevents cell cycle arrest at the G0/G1 phase and suppresses apoptosis in A549 and H1299 cells (Zhang et al., 2016). In particular, RPS27a inhibits cell cycle arrest and apoptosis by regulating the RPL11–MDM2–p53 pathway in lung adenocarcinoma (Li et al., 2022).

Several studies have shown that miR-30a functions as a tumor suppressor in lung cancer by targeting genes such as DNMT3A, SIRT1, and CD73/NT5E (Guan et al., 2017; Wei et al., 2019; Zhu et al., 2017). In line with these reports, our study demonstrated that downregulation of miR-30a in TB-derived exosomes contributes to lung cancer progression by inducing the upregulation of ribosome-related genes, including RPS27 and RPS15A.

Altered expression of miR-185 has been reported in patients with tuberculosis TB and LC (Kaushik et al., 2021; Lei et al., 2018). Consistent with previous reports, our study confirmed the significant upregulation of miR-185 in the TB-associated LC group. Network analysis predicted an association with the NDUF gene family and the RHOA gene. However, significant regulation of these genes was not observed in our experimental model. In contrast, further *in vitro* experiments shown that the inhibition of miR-185 led to increased expression of RPS27. RPS27 is a known factor that promotes lung cancer progression (Li et al., 2022). These findings suggest that certain components of TB-derived exosomes may function as a localized compensatory response aimed at counteracting tumor progression.

Cytochrome c oxidase subunit 7A (COX7A) refers to a family of genes encoding subunits of complex IV of the mitochondrial respiratory chain, which is involved in oxidative phosphorylation (Zhao L. et al., 2019). Some studies suggest that downregulation of COX7A2 is associated with poor prognosis in non-small cell lung cancer (Mishra et al., 2017; Zhao L. et al., 2019). In addition, COX7A1 has been reported to enhance the sensitivity of lung cancer cells to cystine deprivation–induced ferroptosis by regulating the TCA cycle and mitochondrial activity (Feng et al., 2022). Accordingly, our finding that downregulation of miR-224 in TB-associated exosomes leads to COX7A upregulation suggests the potential applicability of ferroptosis inducers as a novel therapeutic strategy in TB-associated lung cancer.

This study has several limitations. First, the sample size for both the *in vivo* and *in vitro* experiments was relatively small, which may limit the generalizability of our findings. Although biological consistency of the results was considered through various analyses, we believe that the possibility of statistical errors arising from the limited data size may still be present in our findings. This issue, together with the statistical shortcomings associated with the use of an outdated pipeline, may hinder accurate quantification of expression levels and thereby compromise the reliability of the results. In the selection of DEMs with a limited sample size, the small number of biological replicates can be a primary factor reducing the reliability of the identified miRNAs, despite the application of methods to mitigate this problem. To address this limitation, we validated the association of miRNA regulation through *in vitro* experiments, however, further studies with a larger sample size are required. Nevertheless, these findings provide clues for elucidating the mechanisms of tuberculosis-related carcinogenesis by experimentally confirming the indirect effects of miRNA regulation. Second, although PEs from patients with TB were used for exosomal miRNA analysis, lung tissue samples could not be obtained from patients with TB-associated lung cancer. To address this limitation, we employed a xenograft mouse model in which human lung cancer cells were co-injected with TB patient–derived exosomes. Certainly, the use of xenograft model for the analysis of TB-associated lung cancer-related genes leaves unresolved limitations, including whether the findings can be reproduced in actual patient tissue samples and the issue of mouse contamination inherent to xenograft models, both of which require further investigation. Also, the limitation of xenograft experiment is that the use of intratumoral injection does not fully reflect the physiological route of exosome delivery from the pleural space to tumors and a matched exosome control (e.g., transudate-derived exosomes) was not incorporated. Therefore, our findings should be interpreted as demonstrating potential tumor-promoting effects rather than exact *in vivo* behavior. Finally, although we experimentally validated the proposed miRNA–mRNA regulatory networks, further studies with larger clinical cohorts and additional mechanistic experiments are needed to confirm the identified biomarkers and therapeutic targets in TB-associated lung cancer.

In conclusion, our integrated *in silico* analysis and experimental validation identified multiple miRNA–mRNA regulatory pathways that may contribute to the carcinogenesis of TB-associated lung cancer. These findings provide new insights into the molecular mechanisms underlying the miRNA–mRNA regulatory network in the pathogenesis of this disease.

## Data Availability

The data underlying this study cannot be shared publicly due to privacy restrictions. Researchers wishing to access the data should direct their inquiries to the corresponding authors.

## References

[B1] Abdeahad H. Salehi M. Yaghoubi A. Aalami A. H. Aalami F. Soleimanpour S. (2022). Previous pulmonary tuberculosis enhances the risk of lung cancer: systematic reviews and meta-analysis. Infect. Dis. 54 (4), 255–268. 10.1080/23744235.2021.2006772 34807803

[B2] Ashenafi S. Brighenti S. (2022). Reinventing the human tuberculosis (TB) granuloma: learning from the cancer field. Front. Immunol. 13, 1059725. 10.3389/fimmu.2022.1059725 36591229 PMC9797505

[B3] Balsa E. Marco R. Perales-Clemente E. Szklarczyk R. Calvo E. Landazuri M. O. (2012). NDUFA4 is a subunit of complex IV of the Mammalian electron transport chain. Cell. Metab. 16 (3), 378–386. 10.1016/j.cmet.2012.07.015 22902835

[B4] Bindea G. Galon J. Mlecnik B. (2013). CluePedia Cytoscape plugin: pathway insights using integrated experimental and *in silico* data. Bioinformatics 29 (5), 661–663. 10.1093/bioinformatics/btt019 23325622 PMC3582273

[B5] Boukouris S. Mathivanan S. (2015). Exosomes in bodily fluids are a highly stable resource of disease biomarkers. Proteomics Clin. Appl. 9 (3-4), 358–367. 10.1002/prca.201400114 25684126 PMC5502131

[B6] Bray F. Laversanne M. Sung H. Ferlay J. Siegel R. L. Soerjomataram I. (2024). Global cancer statistics 2022: GLOBOCAN estimates of incidence and mortality worldwide for 36 cancers in 185 countries. CA A Cancer Journal Clinicians 74 (3), 229–263. 10.3322/caac.21834 38572751

[B7] Cabrera-Sanchez J. Cuba V. Vega V. Van der Stuyft P. Otero L. (2022). Lung cancer occurrence after an episode of tuberculosis: a systematic review and meta-analysis. Eur. Respiratory Review 31 (165), 220025. 10.1183/16000617.0025-2022 35896272 PMC9724897

[B8] Chen Y. Wang X. (2020). miRDB: an online database for prediction of functional microRNA targets. Nucleic Acids Res. 48 (D1), D127–D131. 10.1093/nar/gkz757 31504780 PMC6943051

[B9] Chen Z. Wang T. Liu Z. Zhang G. Wang J. Feng S. (2015). Inhibition of autophagy by MiR-30A induced by mycobacteria tuberculosis as a possible mechanism of immune escape in human macrophages. Jpn. J. Infect. Dis. 68 (5), 420–424. 10.7883/yoken.JJID.2014.466 25866116

[B10] Dacosta N. A. Kinare S. G. (1991). Association of lung carcinoma and tuberculosis. J. Postgrad. Med. 37 (4), 185–189. Available online at: https://www.ncbi.nlm.nih.gov/pubmed/1841965 (Accessed May 8, 2026). 1841965

[B11] Dobin A. Davis C. A. Schlesinger F. Drenkow J. Zaleski C. Jha S. (2013). STAR: ultrafast universal RNA-seq aligner. Bioinformatics 29 (1), 15–21. 10.1093/bioinformatics/bts635 23104886 PMC3530905

[B12] Feng Y. Xu J. Shi M. Liu R. Zhao L. Chen X. (2022). COX7A1 enhances the sensitivity of human NSCLC cells to cystine deprivation-induced ferroptosis *via* regulating mitochondrial metabolism. Cell. Death and Disease 13 (11), 988. 10.1038/s41419-022-05430-3 36418320 PMC9684511

[B13] Friedländer M. R. Mackowiak S. D. Li N. Chen W. Rajewsky N. (2012). miRDeep2 accurately identifies known and hundreds of novel microRNA genes in seven animal clades. Nucleic Acids Research 40 (1), 37–52. 10.1093/nar/gkr688 21911355 PMC3245920

[B14] Guan Y. Rao Z. Chen C. (2017). miR-30a suppresses lung cancer progression by targeting SIRT1. Oncotarget 9 (4), 4924–4934. 10.18632/oncotarget.23529 29435152 PMC5797023

[B15] Heuvers M. E. Aerts J. G. Hegmans J. P. Veltman J. D. Uitterlinden A. G. Ruiter R. (2012). History of tuberculosis as an independent prognostic factor for lung cancer survival. Lung Cancer 76 (3), 452–456. 10.1016/j.lungcan.2011.12.008 22226628

[B16] Hong S. Mok Y. Jeon C. Jee S. H. Samet J. M. (2016). Tuberculosis, smoking and risk for lung cancer incidence and mortality. Int. Journal Cancer 139 (11), 2447–2455. 10.1002/ijc.30384 27521774

[B17] Huang Z. Yao F. Liu J. Xu J. Guo Y. Su R. (2020). Up-regulation of circRNA-0003528 promotes Mycobacterium tuberculosis associated macrophage polarization *via* down-regulating miR-224-5p, miR-324-5p and miR-488-5p and up-regulating CTLA4. Aging (Albany NY) 12 (24), 25658–25672. 10.18632/aging.104175 33318319 PMC7803570

[B18] Jiang H. Lei R. Ding S. W. Zhu S. (2014). Skewer: a fast and accurate adapter trimmer for next-generation sequencing paired-end reads. BMC Bioinforma. 15, 182. 10.1186/1471-2105-15-182 24925680 PMC4074385

[B19] Kang H.-J. Yun S. Shin S.-H. Youn D. H. Son G.-H. Lee J. J. (2024). Tuberculous pleural effusion-derived exosomal miR-130b-3p and miR-423-5p promote the proliferation of lung cancer cells *via* cyclin D1. Int. J. Mol. Sci. 25 (18), 10119. 10.3390/ijms251810119 39337604 PMC11431986

[B20] Kaushik A. C. Wu Q. Lin L. Li H. Zhao L. Wen Z. (2021). Exosomal ncRNAs profiling of mycobacterial infection identified miRNA-185-5p as a novel biomarker for tuberculosis. Briefings Bioinforma. 22 (6), bbab210. 10.1093/bib/bbab210 34169968

[B21] Kozomara A. Birgaoanu M. Griffiths-Jones S. (2019). miRBase: from microRNA sequences to function. Nucleic Acids Research 47 (D1), D155–D162. 10.1093/nar/gky1141 30423142 PMC6323917

[B22] Langmead B. Trapnell C. Pop M. Salzberg S. L. (2009). Ultrafast and memory-efficient alignment of short DNA sequences to the human genome. Genome Biology 10, 1–10. 10.1186/gb-2009-10-3-r25 19261174 PMC2690996

[B23] Lei L. Chen C. Zhao J. Wang H. Guo M. Zhou Y. (2017). Targeted expression of miR-7 operated by TTF-1 promoter inhibited the growth of human lung cancer through the NDUFA4 pathway. Mol. Ther. Nucleic Acids 6, 183–197. 10.1016/j.omtn.2016.12.005 28325285 PMC5363496

[B24] Lei Z. Shi H. Li W. Yu D. Shen F. Yu X. (2018). miR-185 inhibits non-small cell lung cancer cell proliferation and invasion through targeting of SOX9 and regulation of Wnt signaling. Mol. Medicine Reports 17 (1), 1742–1752. 10.3892/mmr.2017.8050 29138830 PMC5780119

[B25] Li H. Zhang H. Huang G. Bing Z. Xu D. Liu J. (2022). Loss of RPS27a expression regulates the cell cycle, apoptosis, and proliferation *via* the RPL11-MDM2-p53 pathway in lung adenocarcinoma cells. J. Exp. and Clin. Cancer Res. 41 (1), 33. 10.1186/s13046-021-02230-z 35073964 PMC8785590

[B26] Liao K.-M. Lee C.-S. Wu Y.-C. Shu C.-C. Ho C.-H. (2023). Prior treated tuberculosis and mortality risk in lung cancer. Front. Med. 10, 1121257. 10.3389/fmed.2023.1121257 37064038 PMC10090669

[B27] Light R. W. Macgregor M. I. Luchsinger P. C. Ball W. C. Jr. (1972). Pleural effusions: the diagnostic separation of transudates and exudates. Ann. Intern Med. 77 (4), 507–513. 10.7326/0003-4819-77-4-507 4642731

[B28] Martin M. (2011). Cutadapt removes adapter sequences from high-throughput sequencing reads. EMBnet. Journal 17 (1), 10–12. 10.14806/ej.17.1.200

[B29] Mathivanan S. Ji H. Simpson R. J. (2010). Exosomes: extracellular organelles important in intercellular communication. J. Proteomics 73 (10), 1907–1920. 10.1016/j.jprot.2010.06.006 20601276

[B30] Mishra N. Timilsina U. Ghimire D. Dubey R. C. Gaur R. (2017). Downregulation of cytochrome c oxidase subunit 7A1 expression is important in enhancing cell proliferation in adenocarcinoma cells. Biochem. Biophysical Res. Commun. 482 (4), 713–719. 10.1016/j.bbrc.2016.11.100 27866983

[B31] Moon S. M. Choi H. Kim S. H. Kang H. K. Park D. W. Jung J. H. (2023). Increased lung cancer risk and associated risk factors in tuberculosis survivors: a Korean population-based study. Clin. Infect. Dis. 77 (9), 1329–1339. 10.1093/cid/ciad373 37345907 PMC10640693

[B32] Mukhtar F. Guarnieri A. Brancazio N. Falcone M. Di Naro M. Azeem M. (2024). The role of *Mycobacterium tuberculosis* exosomal miRNAs in host pathogen cross-talk as diagnostic and therapeutic biomarkers. Front. Microbiology 15, 1441781. 10.3389/fmicb.2024.1441781 39176271 PMC11340542

[B33] Nail H. M. Chiu C.-C. Leung C.-H. Ahmed M. M. Wang H.-M. D. (2023). Exosomal miRNA-mediated intercellular communications and immunomodulatory effects in tumor microenvironments. J. Biomed. Sci. 30 (1), 69. 10.1186/s12929-023-00964-w 37605155 PMC10440907

[B34] Nalbandian A. Yan B. Pichugin A. Bronson R. Kramnik I. (2009). Lung carcinogenesis induced by chronic tuberculosis infection: the experimental model and genetic control. Oncogene 28 (17), 1928–1938. 10.1038/onc.2009.32 19330024

[B36] Peng Y. Zhu X. Gao L. Wang J. Liu H. Zhu T. (2022). *Mycobacterium tuberculosis* Rv0309 dampens the inflammatory response and enhances mycobacterial survival. Front. Immunol. 13, 829410. 10.3389/fimmu.2022.829410 35281073 PMC8907127

[B37] R Core Team (2024). R: A Language and Environment for Statistical Computing. Vienna, Austria: R Foundation for Statistical Computing. Available online at: https://www.R-project.org/ (Accessed May 8, 2026).

[B38] Robinson M. D. McCarthy D. J. Smyth G. K. (2010). edgeR: a bioconductor package for differential expression analysis of digital gene expression data. Bioinformatics 26 (1), 139–140. 10.1093/bioinformatics/btp616 19910308 PMC2796818

[B39] Shannon P. Markiel A. Ozier O. Baliga N. S. Wang J. T. Ramage D. (2003). Cytoscape: a software environment for integrated models of biomolecular interaction networks. Genome Research 13 (11), 2498–2504. 10.1101/gr.1239303 14597658 PMC403769

[B40] Sheikhpour M. Abolfathi H. Karimipoor M. Movafagh A. Shahsavani M. (2021). The common miRNAs between tuberculosis and non-small cell lung cancer: a critical review. Tanaffos 20 (3), 197–208. 35382078 PMC8978040

[B41] Sinigaglia A. Peta E. Riccetti S. Venkateswaran S. Manganelli R. Barzon L. (2020). Tuberculosis-associated microRNAs: from pathogenesis to disease biomarkers. Cells 9 (10), 2160. 10.3390/cells9102160 32987746 PMC7598604

[B42] Spinelli S. V. Fernández R. d. V. Zoff L. Bongiovanni B. Díaz A. D'Attilio L. (2017). miR-30c is specifically repressed in patients with active pulmonary tuberculosis. Tuberculosis 105, 73–79. 10.1016/j.tube.2017.04.004 28610790

[B43] Szklarczyk D. Gable A. L. Lyon D. Junge A. Wyder S. Huerta-Cepas J. (2019). STRING v11: protein-protein association networks with increased coverage, supporting functional discovery in genome-wide experimental datasets. Nucleic Acids Res. 47 (D1), D607–D613. 10.1093/nar/gky1131 30476243 PMC6323986

[B44] Trapnell C. Williams B. A. Pertea G. Mortazavi A. Kwan G. van Baren M. J. (2010). Transcript assembly and quantification by RNA-seq reveals unannotated transcripts and isoform switching during cell differentiation. Nat. Biotechnol. 28 (5), 511–515. 10.1038/nbt.1621 20436464 PMC3146043

[B45] Trapnell C. Hendrickson D. G. Sauvageau M. Goff L. Rinn J. L. Pachter L. (2013). Differential analysis of gene regulation at transcript resolution with RNA-seq. Nat. Biotechnol. 31 (1), 46–53. 10.1038/nbt.2450 23222703 PMC3869392

[B46] Wang W. Nag S. Zhang X. Wang M. H. Wang H. Zhou J. (2015). Ribosomal proteins and human diseases: pathogenesis, molecular mechanisms, and therapeutic implications. Med. Research Reviews 35 (2), 225–285. 10.1002/med.21327 25164622 PMC4710177

[B47] Wang J. Ni J. Beretov J. Thompson J. Graham P. Li Y. (2020). Exosomal microRNAs as liquid biopsy biomarkers in prostate cancer. Crit. Reviews Oncology/hematology 145, 102860. 10.1016/j.critrevonc.2019.102860 31874447

[B48] Wang Q. Wang G. Niu L. Zhao S. Li J. Zhang Z. (2021). Exosomal MiR-1290 promotes angiogenesis of hepatocellular carcinoma *via* targeting SMEK1. J. Oncol. 2021, 6617700. 10.1155/2021/6617700 33564307 PMC7864765

[B49] Wang L. Xiong Y. Fu B. Guo D. Zaky M. Y. Lin X. (2022). MicroRNAs as immune regulators and biomarkers in tuberculosis. Front. Immunol. 13, 1027472. 10.3389/fimmu.2022.1027472 36389769 PMC9647078

[B50] Wei D. Yu G. Zhao Y. (2019). MicroRNA-30a-3p inhibits the progression of lung cancer *via* the PI3K/AKT by targeting DNA methyltransferase 3a. OncoTargets Therapy 12, 7015–7024. 10.2147/OTT.S213583 31695416 PMC6717841

[B51] Woo S. J. Kim Y. Jung H. Lee J. J. Hong J. Y. (2021). Tuberculous fibrosis enhances tumorigenic potential *via* the NOX4-Autophagy axis. Cancers (Basel) 13 (4), 687. 10.3390/cancers13040687 33567693 PMC7916030

[B52] Woo S. J. Kim Y. Kang H.-J. Jung H. Youn D. H. Hong Y. (2024). Tuberculous pleural effusion-induced Arg-1+ macrophage polarization contributes to lung cancer progression *via* autophagy signaling. Respir. Res. 25 (1), 198. 10.1186/s12931-024-02829-8 38720340 PMC11077851

[B35] World Health Organization (2021). Global Tuberculosis Report. Available online at: https://www.who.int/teams/global-tuberculosis-programme/tb-reports/global-tuberculosis-report-2021 (Accessed May 8, 2026).

[B53] Wu Y. Sun Q. Dai L. (2017). Immune regulation of miR-30 on the mycobacterium tuberculosis-induced TLR/MyD88 signaling pathway in THP-1 cells. Exp. Ther. Med. 14 (4), 3299–3303. 10.3892/etm.2017.4872 28912881 PMC5585754

[B54] Yu G. (2024). Thirteen years of clusterProfiler. Innov. (Camb) 5 (6), 100722. 10.1016/j.xinn.2024.100722 39529960 PMC11551487

[B55] Zhang Y. Zhang G. Li X. Li B. Zhang X. (2016). The effect of ribosomal protein S15a in lung adenocarcinoma. PeerJ 4, e1792. 10.7717/peerj.1792 26989627 PMC4793315

[B56] Zhao L. Chen X. Feng Y. Wang G. Nawaz I. Hu L. (2019a). COX7A1 suppresses the viability of human non‐small cell lung cancer cells *via* regulating autophagy. Cancer Med. 8 (18), 7762–7773. 10.1002/cam4.2659 31663688 PMC6912042

[B57] Zhao Z. Hao J. Li X. Chen Y. Qi X. (2019b). MiR‐21‐5p regulates mycobacterial survival and inflammatory responses by targeting Bcl‐2 and TLR4 in mycobacterium tuberculosis‐infected macrophages. FEBS Letters 593 (12), 1326–1335. 10.1002/1873-3468.13438 31090056

[B58] Zhou X. Liao W.-J. Liao J.-M. Liao P. Lu H. (2015). Ribosomal proteins: functions beyond the ribosome. J. Molecular Cell Biology 7 (2), 92–104. 10.1093/jmcb/mjv014 25735597 PMC4481666

[B59] Zhou Q. Li X. Zhou H. Zhao J. Zhao H. Li L. (2024). Mitochondrial respiratory chain component NDUFA4: a promising therapeutic target for gastrointestinal cancer. Cancer Cell. Int. 24 (1), 97. 10.1186/s12935-024-03283-8 38443961 PMC10916090

[B60] Zhu J. Zeng Y. Li W. Qin H. Lei Z. Shen D. (2017). CD73/NT5E is a target of miR-30a-5p and plays an important role in the pathogenesis of non-small cell lung cancer. Mol. Cancer 16 (1), 34. 10.1186/s12943-017-0591-1 28158983 PMC5291990

[B61] Zhu T. Liu H. Su L. Dawood A. Hu C. Chen X. (2021). Identification of unique key miRNAs, TFs, and mRNAs in virulent MTB infection macrophages by network analysis. Int. J. Mol. Sci. 23 (1), 382. 10.3390/ijms23010382 35008808 PMC8745702

