# Supplementary material for: Analysis of gene expression patterns modulated by tuberculous pleural effusion–derived exosomal miRNAs in lung cancer
Source: Front Genet. 2026 May 29;17:1828734. doi: 10.3389/fgene.2026.1828734 (PMC13258543; doi:10.3389/fgene.2026.1828734)
Supplement: Supplementary file 5 [file DataSheet1.docx]

Supplementary Material

# Supplementary Figures and Tables

**Supplementary Table 1.** Summary of sequencing data and quality control statistics for mRNA and miRNA libraries.

**Supplementary Table 2.** List of PCR primers used for the experiments

**Supplementary Table 3.** List of all DEMs in TPE-derived exosomes compared with T-derived exosomes.

**Supplementary Table 4.** List of all DEGs in TPE-derived exosome-treated mice compared with PBS-injected control mice.

## Supplementary Figures


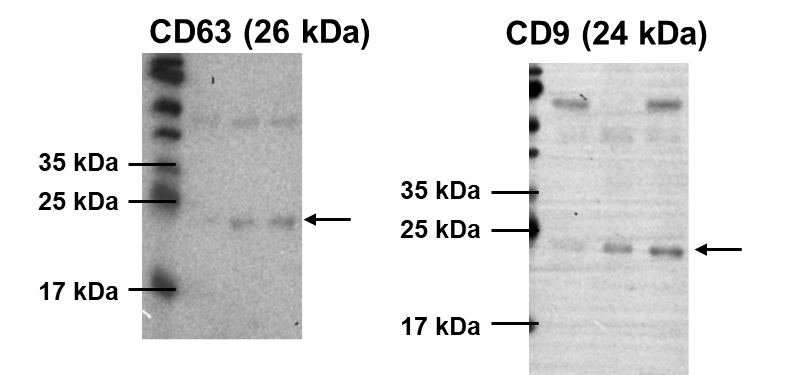


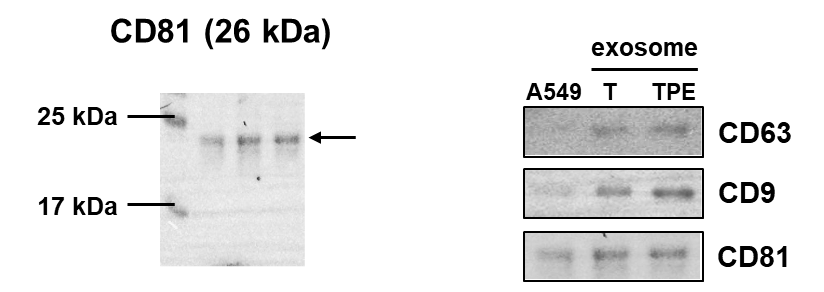


**Supplementary Figure 1.** Original, unprocessed Western blot images showing the expression of exosomal markers (CD63, CD9, and CD81) in exosomes isolated from tuberculous pleural effusion (TPE) and transudate samples.
